# Collectanea

**Published:** 1827-05

**Authors:** 


### COLLECTANEA.

Floriferis ut apes in saltibus omnia libant,

Omnia nos, itidem, depascimur aurea dicta.

### PHYSIOLOGY.

*Cupping Glasses applied over Vaccine Punctures.*—At some of the late meetings of the Academie Royale de Medecine, the subject of preventing the absorption of vaccine matter by means of cupping glasses, occupied the attention of the Society. M. Itard vaccinated three children, one of whom had previously been vaccinated; the second was doubtful; and the third had never had either small-pox or been vaccinated. In all, the punctures were made on the shoulders. They failed completely in the two first; but in the third there appeared, on the side which had not been covered with the cupping glass, five vaccine pustules, and on the other side one pustule only, which had been purposely left uncovered by the glasses.

M. Bousquet reported that he had been making similar experiments on vaccine pustules, and that the application of the cupping glasses in no wise arrested the development of the pustule. (*Revue Medicale.*)

*Cephalo-Spinal Fluid.*—Two things often puzzled us when examining the bodies of the dead,—the disproportion of size between the spinal marrow and the canal in which it is contained; and the fact that, in almost all cases, there was more or less serous effusion (as it is called) at the base of the skull, and in the vertebral canal. In a late sitting of the Royal Institute of Paris, M. Magendie read a Memoir on the Fluid found in the Cranial and Spinal Cavities of Man and Mammiferous Animals, to which fluid he has given the epithet *Cephalorachidien.* This eminent physiologist commences with an estimate of the total amount of this fluid, which he found to vary, in the adult and healthy subject, from two to five ounces. Among other uses, M. Magendie considers the cephalo-spinal fluid to serve the purpose of keeping, in a proper degree of plenitude, the cavities of the cranium and spine. In old age, the shrinking up of the brain and spinal marrow (not sufficiently observed by physiologists) causes a greater space for the accumulation of this fluid, and is thus inimical to the sustenance of life. In corroboration of this opinion, M. Magendie has observed that, in the old and emaciated females who die at the Salpetriere, the quantity of this cephalo-spinal fluid is very considerable. Numerous experiments on animals have demonstrated that this same liquid, when drained off artificially, is very quickly reproduced, like the humours of the eye. The effect which this artificial evacuation produces in animals, is a state of numbness and hebetude, which continues till the reproduction of the liquor. In two instances he found the evacuation induce a state of agitation and violence in the animals, which resembled hydrophobia, and lasted for three or four days. An artificial augmentation of the cephalo-rachidien liquor causes pressure on the brain and spinal marrow, and determines paralysis. The disease known by the name of spina bifida consists, according to M. Magendie, of a kind of hernia of the membranes which contain the said fluid. By pressure of the spina bifida, in children, he has produced the same phenomena which were exhibited by animals, where injections had been used to augment the quantum of this fluid. The temperature of the cerebro-spinal liquid was found to be under that of the blood, by some degrees. M. Magendie has produced in animals a tremor and a momentary paralysis, by drawing off this liquor, suffering it to cool, and then injecting again the same fluid. These symptoms appeared to last till the fluid had regained its natural temperature. If, after the evacuation of the fluid, the same was injected again of the natural temperature, no such symptoms occurred. The motion of the head on the trunk of the animal produced a wave or agitation of the fluid in the spine.—M. Magendie is of opinion that the contact of this fluid plays an important part in the development of electricity, and has undertaken a series of experiments, with the view of determining this point. All physiologists now agree in believing that a certain quantity of halitus, or watery vapour, in the ventricles of the brain is consistent with health. M. Magendie has proved, to his own satisfaction at least, that a communication exists naturally between the lateral and the other two ventricles of the brain, and between the fourth ventricle and sub-arachnoid cavity of the spine. This communication, he says, is established by an aperture between the two posterior cerebellic arteries, and is at least three lines in diameter. M. Magendie proposes to call this aperture " entrée des cavitiés du cerveau," or " entrée des ventricules cerebraux."

The communication being thus established between the ventricles of the brain and the spinal canal, M. Magendie avers that, in all morbific affections of the brain, as acute and chronic hydrocephalus, in which there is an undue dilatation of one or more ventricles, these "entrées," or communications, become very much enlarged. The experiment of pressure on a spina bifida affecting the head, proves the communication in the living body. The following experiment was made by M. Magendie:—He injected four ounces of ink, very gently, into the lower portion of the vertebral canal. This was sufficient to blacken, not only the whole surface of the brain, but the internal surfaces of all the ventricles. Whenever pressure was made on the spinal envelopes, a fresh quantity was pushed into the third ventricle.

In more than fifty bodies of people who had died without any cerebral affection, M. Magendie found from half an ounce to two ounces of fluid in the ventricles of the brain. Care should be taken, however, that the body be not placed in a position to favour the draining of the ventricular fluid into the spinal canal. This eminent physiologist is of opinion that, if the cephalo-rachidien liquor amount to more than two ounces, certain morbid phenomena will be the result, especially serous apoplexy. M. Magendie terminates his Memoir with these words:—" Is it not remarkable that those parts of the brain called by the old anatomists *valvule, aqueduct, bridge*, should have precisely the uses which their names import? It is thus that the *valve of Vieussens*, or valvula magna cerebelli, performs the office of a valve (*soupage*), and opposes the exit of fluid from the fourth ventricle. Never did a part better merit its name than the *aqueduct of Sylvius*, since, according to the experiments which I have made, this canal transports the fluid, sometimes from the ventricles to the spine, sometimes from the spine to the head. Finally, that part which has been termed a *pons* is, in fact, an arcade of medullary matter, placed over the current of fluid which traverses the aqueduct." (*Med. Chir. Review*, from *Revue Medicale.*)

---

### PATHOLOGY.

*Identity of the Vaccine and Varioloid Virus.*—M. Kergaradec read a letter which he had received from M. Guillon, an old naval surgeon, vaccinator of the Canton of St. Pol de Leon. This surgeon having to oppose an epidemic small-pox, and having no vaccine matter, took from a girl, fifteen years old, who had been vaccinated, the virus of a varioloid pustule, on the fifth day of the eruption, and inoculated with it a child at the breast. What was his surprise to see ten *superb* vaccine pustules result from this inoculation! Wishing to verify so singular a fact, and fearful of allowing himself to be imposed upon by appearances, he inoculated forty-two children with the matter of these pustules arrived at their ninth day, and these all had the vaccine; these again furnished in their turn virus to inoculate one hundred others, and the operation, performed in the presence of the authorities of the place and several professional men, was followed by the same result. Lastly, M. Guillon, wishing to renew his experiment, inoculated, with the same success, ten other persons with the matter of varioloid pustules, from ten students of the College of St. Pol de Leon, who had been formerly vaccinated.

A later letter announces that numerous experiments confirm the identity of the varioloid and the vaccine virus. The first appears to have even more energy than the second; it acts as a preservative like the second, since individuals inoculated with it had with impunity exposed themselves to the contagion of small-pox. *(Revue Medicale.)*

*Abdominal Tumor.*—M. Lisfranc mentioned the case of a lady who had a moveable tumor in the belly, and which had so distended the integuments of the abdomen, that her extraordinary figure was the jest of every body. All the surgeons who were consulted dreaded any operation for her relief. One day this woman, in straining to pass a little urine, passed at the same time pieces of yellowish mucus, and the size of her belly sensibly subsided. But, during the discharge of these matters, there came on long-continued syncopes, and at short intervals. M. Lisfranc being called, attributed these syncopes to the void made in the abdomen, and he placed the patient in such a position that the inferior part of the pelvis was raised. The discharge ceased, and she regained her strength. However, from time to time the patient was placed so as to facilitate the discharge, and in a month the belly had its natural size. At present there can only be felt in the left flank a hard moveable tumor, the size of a fist, caused by the thickening of the cyst, which has for two years and a half remained in the same state, and continues to furnish by the bladder a small quantity of white matter. This lady has got fat, and enjoys in other respects good health. *(Ibid.)*

*On two new Kinds of Urinary Gravel.*—These new varieties of calculi were observed by M. Magendie. The first occurred with a man of rank, a lover of good eating, who, being in circumstances in which he gave way to his inclinations, thought it right to eat each morning a large plateful of sorrel. After following this plan for more than a year, severe pains were felt in the loins and ureters, and shortly after a calculus was voided, six or seven lines in length, and two in width. It was hard, had an orange colour, and, being analysed, was found to consist of oxalate of lime nearly pure. The oxalic acid introduced into the system by the sorrel was evidently the cause of this calculus, and an effectual remedy was found in change of diet.

The second kind of gravel was of much more uncertain origin. In this disease (as yet undescribed) the saline deposit of the urine assumed two forms,—being sometimes a fine white powder, mixed with a large quantity of small hairs, varying in length from a line to an inch or more; and sometimes, on the contrary, forming white pieces, of unequal and irregular form, having no great degree of consistency, and crushing between the fingers. The fragments did not, however, separate entirely, but adhered together by means of a multitude of small hairs like those described, which, being mixed with, made a part of the mass.

Maceration separates these hairs from both varieties of the hairy gravel, as they have been named. They are then found to differ but little from ordinary hair, except in being finer, and of a grey cinder colour. They are so numerous that the smallest fragments exhibit their extremities, and in certain instances the surface of the stone is visibly covered with them. The accompanying matter being analysed, was found to be phosphate of lime, united to a small quantity of phosphate of magnesia, and uric acid.

Each of these varieties has been observed by M. Magendie but once; the patient from whom the first kind came, rendered an enormous quantity daily. The phosphate of lime common to both varieties is, according to M. Magendie, a result of the excessive use of animal food: as to the origin of the hairs, he does not even form a conjecture. The formation of these calculi was readily prevented by prescribing an almost exclusive regimen of vegetable food and alkalis. (*Journal of Science*, from *Bull. Univ.*)

---

### PRACTICAL MEDICINE.

*Artemisia Vulgaris in Epilepsy.*—The German practitioners have much confidence in this remedy in cases of Epilepsy and Catalepsy, and some cases lately recorded afford additional evidence of its power over these formidable and usually unmanageable complaints. It is recommended to be given in powder, in preference either to the decoction or infusion. GRAFE has lately recorded two cases in which it was successfully administered. It is considered necessary to continue the remedy for some time after the cessation of the paroxysms. (*Journal für Chirurgie und Augenheilkunde.*)

*Obesity.*—A remarkable case of obesity is recorded in the last Number of GRAFE's Journal für Chirurgie, which was radically cured by bleeding, the administration of purgatives, abstinence from animal food, and the use of iodine. During the exhibition of the latter remedy, the patient rapidly decreased in size. It was given in doses of twenty drops of the tincture four times a-day. One grain of iodine dissolved in one drachm of alcohol, is the strength of the tincture employed by the German practitioners. (*Ibid.*)

*Treatment of Pulmonary Complaints.*—In the Clinique of M. le Professeur RECAMIER, the Hydrocyanic Acid has been tried in a dozen patients who showed symptoms of chronic pulmonary catarrh, or advanced phthisis. He began by two, three, or four drops of the acid in four ounces of a simple decoction, and the patient took a spoonful of this every two or three hours. The dose has never been greater than six drops. In three of the patients, no effects were visible: the different functions did not appear to be at all influenced by it. With the greater number, however, it caused warmth, with a sense of constriction at the throat, and heat of stomach; and in some cases colic, more or less severe. In one phthisical case it was necessary to discontinue it, as it caused violent diarrhœa. The urine and the cutaneous secretions did not appear changed by it; the circulation, the sensorial and intellectual functions, were not much influenced. In half of the patients who used the acid, the cough was decidedly diminished, as well as the dyspnœa and difficulty of expectorating. The sputa were not changed. The patients passed better nights. In one young man the cough ceased entirely, as well as the fever; but in the beginning of winter he had a relapse, and sunk under it. With these patients, soothing and other means had failed.

According to these experiments, it appears that the hydrocyanic acid acts chiefly on the digestive canal, and particularly when given in large doses. Indeed, in many of the cases where colic was produced, the medicine had been, by mistake, taken in too large doses.

These facts confirm the observations of M. J. BRUCHENEL, who, in recommending this acid in pulmonary catarrh, says it should not be used till the inflammatory disposition is subdued by depletion. One can easily imagine that the digestive canal then becomes less irritable, and can bear larger doses,—even, as he says, to the extent of six or seven drops, without any bad consequences.

Several cases of recent Hæmoptysis have been treated with Nitras Potassæ, either alone or in combination with conserve of roses. The dose was from a drachm to half an ounce. It caused neither colic nor diarrhœa; the patients complained only of the acrid taste and sense of heat in the throat. The urine was augmented in quantity; and the spitting of blood was first diminished, and then entirely stopped in a few days. In one patient with every symptom of incipient phthisis, the spitting ceased and reappeared several times successively. Although the dose was from the first half an ounce of the nitre, incorporated with a syrup, the patient felt no inconvenience from it. *(Revue Medicale.)*

*Intermittents.*—In the clinical practice at the Hôtel Dieu, M. RECAMIER is sometimes in the habit of using the following mixture to check the accession of a paroxysm of ague:—℞. Vini ℥iv.; Sulph. Quinæ gr. xij.; Syrupi Menthæ ℥v. *(Ibid.)*

*Severe Case of Singultus.*—Aleindon, a servant, ætatis forty, a native of Senegal, but who had lived from infancy in France, generally had good health; twelve years ago had had a severe attack of singultus, which was cured by the employment of Hoffman's Anodyne. On the 30th November, 1826, without any known cause, he was seized with shivering, which was soon accompanied with singultus and dyspnœa. The respiration became shorter, and the singultus almost constant. No fever.

1st, 2d, and 3d of December.—The symptoms much aggravated.

5th.—He was taken to the Hôtel Dieu. The difficulty of breathing was extreme.—Sinapisms were applied to the feet, without relief.

6th.—The following were the symptoms: Considerable dyspnœa, corresponding to the almost continual and violent hiccoughs. The patient could not lie down on either side; there was a little cough, accompanied with a trifling mucous expectoration; the pulsations of the heart were regular; the pulse full, and scarcely accelerated; the tongue moist and whitish.—A potion containing five drops of Hydrocyanic Acid, was given; and dry cupping to the epigastrium.

7th.—Breathing worse; the singultus more severe; face much changed; the superior extremities becoming cold; pulse more frequent.—An abundant bleeding was practised, with relief; an antispasmodic draught given.

8th.—Symptoms better, particularly the singultus.—Same treatment, except the bleeding; and a bandage was passed tightly round the body.

On the 10th, the patient continued to improve; but it was not till the 11th that the singultus quite left him, and with it the dyspnœa. It was, however, necessary to use several blisters before the organs of respiration were restored to a healthy state. *(Ibid.)*

*Tartar Emetic.*—M. VELPEAU, in his account of the clinical practice at the Hospital de Perfectionnement, takes notice of the opinion of the Broussaists, that a gastro-enterite always precedes or accompanies erysipelas, and that therefore the emetic plan, so much recommended by DESAULT, is the worst possible; and that only blood-letting, and emollients internally, are to be used. Now, M. Velpeau is of opinion that, although it is sometimes good practice to bleed and apply leeches where there are symptoms of congestion or internal inflammation, yet that the other plan ought by no means to be abandoned when it is indicated. He relates several cases of decided benefit from the use of Tartar Emetic, given in doses to excite vomiting.

He also relates several cases of Rheumatism, where a trial was given to the Tartar Emetic, in doses of twenty and thirty grains. His opinion of the effects of this remedy, after witnessing thirty cases of disease in which it was used, is that it in none produced the least amelioration. In one case, in private practice, there appeared to result some benefit from its use.

Mme. H. suffered severe pain in the legs for fifteen days, in consequence of fatigue, when suddenly these parts swelled and became extremely painful; the pulse was strong and very frequent.—Bleeding to ten ounces.

Next morning, all her limbs were affected; swelling and redness of skin considerable, especially near the joints. The pain was insupportable.—Sixty leeches were applied.

Third day, her sufferings were increased; the loss of blood had produced much weakness; tongue much coated.—Twelve grains of Tartar Emetic were prescribed, in eight ounces of orange-flower water, to be taken by spoonfuls during twenty-four hours.

Fourth day, the patient was up and walking about her room: there remained only a feeling of being bruised in her limbs. She speedily recovered.

In some of the patients in the hospital, this remedy was sometimes carried to the extent of thirty, forty, and forty-five grains; and it must be confessed that, if it did no good, it never appeared to do any harm. The region of the stomach never became painful; the tongue remained healthy; appêtite generally continued; and if, for the first day or two, nausea and looseness showed themselves, they ceased spontaneously, although the dose of the medicine was augmented. These facts, and many similar, prove at least that the destructive qualities which have been attributed to tartar emetic do not necessarily follow its exhibition even in large doses. *(Ibid.)*

---

### MEDICAL JURISPRUDENCE.

*Medico-legal Report of an Exhumation, made three Years after the Interment.* By Dr. Eugene Delmas.—The following is taken from the Edinburgh Journal of Medical Science, being translated from the *Ephemerides Medicales of Montpellier.*

A Piedmontese, named Bonino, formerly a soldier, aged forty-six years, had retired to a village near Montpellier. He disappeared in 1823, and a report spread that he had gone to Spain; but soon after it was whispered that he had been assassinated by a girl with whom he lived, and a person named Diamont, who was known to have been long intimate with her, and who had married her nine months after the disappearance of Bonino. Two years passed away, however; after which, in 1826, the legal authorities being informed of the rumors spread abroad, caused a search, and a body was found in the garden of the suspected person. In the first place, it was necessary to ascertain if the body was that of Bonino, which could be recognised by one peculiar circumstance,—namely, a sixth finger on the right hand, and a sixth toe on the left foot. I was charged with the examination of the affair, and the following report is the result. Diamont and his wife were tried and condemned by the Court of Assizes at Montpellier, on the 26th August, 1826.

On the 30th April, 1826, at the request of the public administration, we went to the commune of Sussargues, to proceed to the exhumation of a dead body discovered in a garden. The earth being removed, we found, at the depth of eighteen inches, a human skeleton lying on its back. The head, placed towards the north, was slightly bent forward; the lower jaw was separated from the upper. The arms were crossed upon the breast, so that the right passed a little over the left. The ribs, still retaining the form of the thorax, were separated from the sternum, which we found lying on the opposite vertebræ. Some black hair, and a metal button, were imbedded in a moist earthy matter, which covered the anterior surface of the sternum. The vertebral column, unbroken, had retained its relations with the head and pelvis. The inferior extremities, stretched out, and on the same level as the trunk, followed the direction of the axis of the body, and inclined towards each other. The right foot, which alone we saw in its place, was still in the shoe, a little bent on the leg, and inclined to its outer edge; the left had been removed with the shoe, in which we found only a part of it.

The head, removed from its position, was dry in the frontal region, while the occipital region was still moist, and lubrified by a fatty matter, amongst which we found some black hair: attentively observed, it presented to us, at the right external orbitar angle, a deformity arising from an injury long anterior to death, since nature had produced a cure; we thought from this that there had been a cicatrix in this part. Another lesion of the bone existed on the left side of the coronal suture, but it appeared of ancient date. The left temporal bone, especially, fixed our attention: its squamous portion, almost separated from the parietal bone, was divided into three portions by three cracks, which proceeded from the circumference of the bone, and, before the external auditory canal, united to a fourth, which, turning round the base of the zygomatic process, terminated in the glenoid cavity. The form of this fracture, the soundness of the zygomatic arch and mastoid process, induced us to suppose that it was made with a blunt instrument of a small size. From the absence of any apparent operation of nature to effect a cure, from the separation of the osseous pieces, and the oozing which took place through the different points of the fracture, we think that it had taken place at a time very near death. We add even that the injuries we observed were the results of a violent blow, that must have brought on a cerebral commotion, which, without considering other accidents, would instantly deprive the individual of the use of his senses, and every means of defence.

The shoes in which we found the bones of the foot, some pieces of woollen cloth surrounding the vertebræ of the neck, metal and wooden buttons, a knife, of which the blade was folded in the handle, and found at the left side of the breast, some fragments of cloth and velvet, all these inclined us to believe that the body had been buried covered with a part, at least, of its clothes.

Although the time necessary for the complete decomposition of a dead body be very variable, and no exact rule can be established in respect to this, seeing that climate, moistness or dryness of soil, the greater or less depth of graves, circumstances relative to the state or temperament of individuals, all occasion much difference, we endeavoured, nevertheless, to determine how long the skeleton in question had been buried. The general opinion is that, in a temperate climate, when no peculiar circumstance hastes on or delays decomposition, it is completed in three or four years. In comparing the state in which we found the parts at the time of exhumation, with what has been said on this subject, we think we may advance that the body has been buried about three years and a half. We indeed remarked what some authors point out as taking place in the third period, that begins after the third year,—namely, the entire disappearance of gaseous products, the fetid odour replaced by an odour of mouldiness, and merely a remains of earthy, fatty, friable, brownish and black matter. The only soft parts which we found were vertebral ligaments, the composition of which, from being more nearly of the nature of bone, ought to be the last to disappear.

As neither time nor place permitted an attentive examination of the other parts of the skeleton, we put all the bones that we could find into a bag, to which the legal seal was applied.

On the 5th of May, we went to the judges' chamber, to continue the examination of the bones. We found all the vertebræ, the ribs, and bones of the pelvis, which were soon articulated. Wishing to determine to what sex the skeleton belonged, we examined these different parts. By the width of the passages being small compared to the depth of the pelvis, the outlet narrow, cordiform, and terminated in a point forwards, a formation that depends on the direction of the ischia, which in their descent converge much towards each other, by the oval and very elongated form of the obturator holes, we were induced to believe that it belonged to a man. We are confirmed in our opinion by the small separation of the descending rami of the pubis, which had their anterior face directed outwards; whilst in the female it is broad and flat. These circumstances were in relation with the length, the consistence, and development of the bones.

The sex being known, we endeavoured to discover the man's age. The complete development of the bones, that of the processes to which the muscles are attached, and also the jaws; the state of the teeth, the number of which was complete, with the exception of the fourth molar tooth of the right side of the upper jaw, which had been long out, as the alveolar cavity was ossified; and the adjoining teeth had not changed their direction, although they were not supported: these circumstances induced us to say that he had attained his fortieth year. According to the comparative table of Professor Sue, we determined that the height was about five feet five inches.

With the exception of some bones, the extremities were complete, and we articulated the right foot which we had preserved in the shoe. Two sesamoid bones, which are common enough, were the only additional ones that we found. The left foot having been removed in digging, some bones of it were lost. We found only the calcaneum, astragalus, scaphoid, and cuboid, the five metatarsal bones, and three phalanges: this prevented us from articulating it, and ascertaining if there was any thing peculiar. Having separately examined the bones that remained, we found the head of the fourth metatarsal rounded, extending outwards, and presenting a small articular surface, which might have been produced by an extra articulation; but, not having seen in what manner this bone was articulated with the first phalanx, we could not determine if there had been a sixth toe attached to it.

Except some small bones of the carpus, we found all those of the right hand. The fifth bone of the right metacarpus at once attracted our attention: shorter and thicker than that of the other hand, its extremity towards the phalanx separated into two parts, one of which, truly articular, smooth, narrow, rounded and prominent, had the direction of the axis of the bone; whilst the other, corresponding to the cubital edge, formed with it an angle of about eight degrees: not continued so far as the first, it was equally smooth, and presented an articular surface, which differed from it only in its less rounded form. Having tried to articulate the first phalanx of the little finger, it fitted exactly upon the first articular head, and presented upon the side corresponding to the second a depression, the obliquity of which was in relation with the direction that we have assigned to this second surface. This examination of the different parts of the fifth finger leaves no doubt of the nature of the anomaly which it presents: thus we think we may affirm, a sixth finger must necessarily have existed, although we did not find the bones that composed it. The left hand, of which we found all the bones except some bones of the carpus, presented no peculiarity.

Such are the results which we obtained; they conduct us to the following conclusions:

1st. The skeleton which we disinterred had been buried from three years to three years and a half, covered with its clothes.

2d. This skeleton belonged to a man about the age of forty to forty-five years, and of the height of five feet five inches.

3d. This man had six fingers on the right hand, the sixth should have been placed beside the little finger; and, if there existed an additional toe on the foot, which we cannot affirm, its place would be on the left foot, on the outside of the little toe.

4th. Death was occasioned by a violent blow given by a blunt instrument, which fractured the left temporal bone.

The value of anatomy, and the advantages derived from it every day by medicine generally, and especially by medical jurisprudence, cannot be denied. The fact just related is a new and remarkable proof of this. It was this science alone that produced evidence on the trial: by it the skeleton, reproduced with its deformities, placed the victim in presence of his assassins, and fully convinced their judges.

Great anomalies have been observed regarding supernumerary fingers: they have been sometimes seen supported by a sixth metacarpal bone, at other times resting on the fifth of these bones, or on the first phalanx of the little finger; sometimes also, but more rarely, affixed immediately to the radial edge. M. Boyer relates, that he has observed them forming a bifurcation upon the first phalanx of the thumb; and I have myself met with a fact of this nature. It has also been observed that additional fingers are rarely found on both hands. Thus, in some persons there may be on one hand a finger very complete, having its bones and muscles, that permit it to execute easy motions; whilst on the other an imperfect finger exists, but little developed, or perhaps only a mere appendage, that leaves no trace on the bone to which it adheres. The same anomalies exist with regard to additional toes.

The present case presents a new example of these aberrations of nature. Bonino had, on the right hand, a sixth finger, formed of one or several phalanges, and whose articulation with the fifth bone of the metacarpus presented a pretty large surface; but it was on the left foot that a sixth toe was found, although the articular surface of the fifth metatarsal bone was less evident than that of the fifth of the metacarpus. Upon the right foot and left hand, on the contrary, there was nothing: in reality, there might have existed an excrescence during life, but, if so, it was not sufficiently developed for any traces of it to be recognised three years after death.

This fact seemed to me interesting, by the circumstances with which it was attended, as but few such are found in the annals of justice, and I thought it my duty to publish it. I do it with so much more confidence, as after having fulfilled, in this unfortunate affair, the so painful duties of a medical jurist, I had the good fortune to acquire, by the confession of the guilty persons, complete security of conscience, in the certainty that I had not been deceived.

### SURGERY.

*Gonorrhœa.*—Every surgeon must have occasionally lamented the obstinacy of this disease, particularly in its chronic form, when the acute symptoms have passed by; and yet the term Gleet can scarçely be correctly applied. In such cases the Copaiba and Cubebs will frequently fail when given singly, although they may prove effectual if combined. In this country it is not so customary to unite these medicines as in Germany. In the last Number of GRAFE's Journal für Chirurgie, this combination is spoken of as frequently effectual, and in confirmation we may add the result of our own experience. In two or three cases of gonorrhœa, in which the discharge was profuse, but the pain trifling, which had continued for a considerable time, the patients were rapidly cured, after having taken the copaiba and cubebs separately without any effect, by the following mixture:—℞. Bals. Copaiv., Cubebæ, āā ℥ss.; Pulv. Acaciæ ʒiij.; Aq. Cinnam. ℥viij. M. capiat coch. tria magna quater in die.—Sir ASTLEY COOPER frequently prescribes these medicines in this form.

*Mode of Stopping Epistaxis.*—A young man, nineteen years of age, bled from the nose two days so profusely, that he fainted several times. Mineral acids, ice to the nape of the neck, &c. were tried, but without stopping the flow of blood. Dr. BRUNNER was called in on the third day, and he blew up powdered gum arabic through a quill: the hemorrhage ceased directly. (HUFELAND's *Journal.*)

*Tapping the Pericardium.*—Mr. JOWETT, of Nottingham, gives the following account of this operation, which he recently performed.

"The following day she was so much worse in all respects, that it was evident she could not long survive, unless some relief were given. The face, as well as the extremities, had become œdematous—respiration was impossible in any other posture except the erect, and there was much accumulation of mucus in the trachea. The operation having been consented to, I performed it in the following manner, the same afternoon, in the presence of Dr. Manson the consulting physician, Mr. Robert Jowett, my brother and pupil, and the patient's friends. Having made a small incision with a lancet through the integuments, between the fifth and sixth cartilages, exactly half way between the sternal extremity of the sixth rib, and the middle of the ensiform cartilage, I thrust a trocar, which had its canula guarded, so that it could not penetrate further than one inch, directly through the thoracic parietes; as I withdrew the trocar, two or three drops of serum escaped, and before I could adapt the pipe of the syringe apparatus to the canula, a little air was sucked through it, during the act of inspiration. On attempting to work the syringe, no fluid was abstracted; imagining, therefore, that I had not punctured the bag, I again introduced the trocar, but with no better success. Certain of the correctness of my diagnosis, I then determined, with Dr. Manson's concurrence, to make another attempt higher up, where there could be no possibility of missing the pericardium, and I accordingly repeated the same process between the fourth and fifth cartilages, as near the sternum as there was space enough for the instrument to pass. Here the trocar seemed to push something before it, which it did not appear to penetrate, and although, here likewise, I twice introduced the instrument, still no fluid could be sucked out by the syringe.

I left the patient under the impression that I had failed in the operation—and my prognosis was "*death in the course of the night.*" Late in the evening, I found her quite as bad, *but no worse* than before.—On the following morning there was an evident amendment—the breathing was less laborious—she coughed more strongly—pulse 126, fullish—the right leg was less swelled, and she had made *two quarts* of urine in the preceding sixteen hours. In the course of that day she made five or six pints more water—the œdema of the lower extremities decreased rapidly, and the improvement was rapidly progressive in every other respect except the cough.

I now perceived, from weighing all these circumstances, that the operation had really been performed, so far as regarded the penetration of the pericardium by the trocar, but in consequence of the canula having been introduced but a little way, it either had never entered the bag, or else had entered so short a distance, as to slip out again immediately on the withdrawal of the trocar. Acting on this view, I renewed every exertion, and the improvement from that time, for eight succeeding days, was such as to warrant great expectations of ultimate success. Weakness was the most threatening circumstance, and that was attempted to be obviated by tonics, wine, and mild nutritious food.

On the 20th Feb. the sound on percussion was dull, only in a moderate-sized præcordial space, and the heart (examined by the stethoscope) had returned to its natural situation. Feb. 22, she felt better—"not so low nor so faint—rested well in the night—appetite improving—tongue clean—pulse quick—ankles a little swelled." She partook more freely of food. The next day, (Feb. 23,) being the tenth from the performance of the operation, my report ran thus—"has had much pain and soreness under the right ribs since last night, and has, twice, this morning vomited green slimy fluid—bowels rather costive. *Hepatis?*" She sunk exhausted in the course of the afternoon.

At my earnest request, seconded by that of Dr. Manson, whose kindness on this and other occasions call for my warmest acknowledgments, the friends permitted an examination of the body to be made, at which Mr. T. R. Tatham also assisted. The exact situations of the punctures were found to be as before described. The *diaphragm* was drawn very high up into the thorax, so that if a pointed instrument had then been introduced perpendicularly at the place of the lowest operation, it would have been punctured. The *pericardium* adhered externally to the anterior part of the left thoracic parietes, through the medium of a layer of firmish recent lymph; and internally it was found every where adherent to the surface of the heart and large vessels, by means of a similar layer of lymph, which varied much in quantity in different places, being in some parts from one-eighth to one-fourth of an inch thick. The adhesion was moderately firm, and the lymph of a reddish colour. As the cavity of the pericardium was destroyed by this state of things, there was of course no fluid remaining it. The pericardium proved to have been perforated at both the points of operation, and there were two holes at each place, so that every time the trocar was introduced, it had penetrated the bag. On the surface of the right ventricle, opposite the upper or last made puncture, there were two dark spots, which, on examination, proved to be drops of coagulated blood enveloped in the layers of lymph, and which had doubtless come from the wounds of the pericardium, as the surface of the heart was untouched. Both the ventricles and auricles were of the natural size. The muscular structure of the heart was rather flabby, and paler than natural. The edge of the mitral valve, on its auricular surface, was beset with a small ridge of semi-cartilaginous lymph, evidently of recent deposition, although firm and hard.

To this long account I may add, that I have since tried the experiment of puncturing the free portion of the pericardium on the dead body, and I have found, that the point of the trocar readily penetrates the sac; but as soon as the edge of the canula (which of course is always rather larger than the instrument it contains) comes in contact with it, it pushes the pericardium before it, and does not enter it, unless it be introduced to a considerable depth. This explains the supposed failure of the operation in the first instance.

I conclude by observing, that the following propositions appear to me fair practical deductions from the case:—

1st, That the operation of tapping the pericardium may be performed without injuring the heart, or endangering the life of the patient.

2nd, That the operation affords a probable chance of saving life, when all other means have failed.

3rd, That it is proper and justifiable under urgent circumstances. (*Medico-Chirurgical Review.*)

---

### CHEMISTRY.

*Acids discovered in Castor Oil.*—MM. Bussy and Lecanu have obtained three new fatty acids from castor oil: one, which they call *ricinic acid*, is fusible at 72° Fahr.; another, termed *elaiodic acid*, is fluid at several degrees below 32°; and the third they have denominated *margaritic acid;* this crystallizes in fine scales, and is not fusible below 264°. These acids are volatile, more or less soluble in alcohol, and perfectly insoluble in water; and they form salts of very distinct characters, with several bases, and especially with magnesia and oxide of lead.

When castor oil is distilled in a retort in the common way, there are obtained a small quantity of gas, water, and acetic acid, a colourless crystallizable volatile oil, ricinic and elaiodic acids, which condense with the oil in the receiver, and a solid matter which remains in the retort. The quantities of acid and of the volatile oil are nearly equal, and form nearly a third of the oil employed; the solid matter constitutes nearly the remaining two-thirds.

This is a very singular substance: it is of a yellowish-white colour, full of cavities, and somewhat resembling the crumb of new bread. It is insoluble in water, alcohol, æther, the volatile and fixed oils. It is dissolved by the alkalies, with which it forms a kind of soap. It is not decomposed at a high temperature, inflames when exposed to an ignited body, and burns very readily without melting. When, instead of distilling castor oil, it is treated with a solution of potash or soda, it saponifies even more readily than olive oil, and there are formed ricinates, elaiodates, margaritates, and glycerin. No other product appears; the glycerin amounts to about a fifteenth part of the oil, the margaritic acid about one-thousandth, and the remainder is constituted of the other acids. These salts are very soluble in water, and act like ordinary soaps; the smallness of the quantity of margaritic acid will account for its not being found in the product of the distillation. (*Philosophical Magazine*, from *Journ. de Pharm.* Feb. 1827.)

---

### MATERIA MEDICA.

*On the Rhubarb of Commerce;* by Mr. David Don.—It is well known that the plant which yields the rhubarb of commerce has been hitherto involved in much obscurity, and hence there have arisen many discordant opinions, both among botanists and pharmacologists, respecting the species of Rheum which affords this valuable medicinal root. They judged it rightly to be the produce of a species of Rheum, but of what particular species, without authentic materials, it was impossible for them to decide. Linnæus considered it at first as the produce of his Rheum rhabarbarum or undulatum, but he afterwards appears to have altered his opinion in favour of Rheum palmatum; while Pallas, who certainly had better opportunities of gaining correct information on the subject, regarded it as composed chiefly of the roots of Rheum undulatum and compactum. Mr. Sievers, an enterprising assistant of Professor Pallas, and well known by his interesting Letters on Siberia, published in the Nordische Beyträge, was sent by the Empress Catharine II. purposely to try to obtain the true rhubarb plant from its native country; and although, after travelling for seven years in the countries adjacent to that in which it is found, he was unable to effect the object of his mission, yet he obtained sufficient information to convince him that the plant was then unknown to botanists. But it was reserved for Dr. Wallich, the zealous superintendent of the Calcutta Botanic Garden, to set this long-agitated question at rest, by the transmission of seeds and dried specimens of the true rhubarb plant to Europe.

Last spring, Mr. Colebrooke received a quantity of the ripe seeds from Dr. Wallich, and presented a portion of them to Mr. Lambert, who has been so fortunate as to raise a number of plants of this valuable vegetable. The seeds were sown in pots, and by the aid of artificial heat, soon vegetated. The young seedlings were transplanted into separate pots filled with rich earth, and the pots were gradually changed as the plants increased in size. By this treatment, as might well be imagined, the young plants grew vigorously, and, at the end of autumn, the leaves were from fifteen inches to a foot in breadth, and the footstalks nine inches long, with half an inch of diameter. The plant, on examination, proved to be identical with my Rheum australe, from Gosaingsthan in the Himalaya Alps. I find Dr. Wallich calls it Rheum Emodi, a name which I should certainly have adopted, had I been aware of it before the publication of my work. The whole plant is thickly beset with numerous small, bristle-shaped, cartilaginous points, which give it a rough feel. The leaves are of a dull green, and the footstalks are red and deeply furrowed. The native samples I have seen appear to be smaller in all their parts, and the leaves, although flowering specimens, frequently not more than three or four inches broad; the footstalks four inches long, and slender; and the flowering stem not above two feet high. It is curious to observe how well this description accords with what Sievers has given us. The Rheum australe appears to be peculiar to the great table lands of central Asia, between the latitudes of 31° and 40°, where it is found to flourish at an elevation of 11,000 feet above the level of the sea; and there is little doubt, therefore, of its proving perfectly hardy in our own country. Large quantities of the root are annually collected for exportation in the Chinese provinces within the lofty range of the Himalaya. The best is that which comes by way of Russia, as greater care is taken in the selection; and on its arrival at Kiachta, within the Russian frontiers, the roots are all carefully examined, and the damaged pieces destroyed. This is the fine rhubarb of the shops, called improperly Turkey Rhubarb. (*Edinburgh New Philosophical Journal.*)

---

### MISCELLANEOUS.

*Poisoned Sugar-Plums.*—It appears that the noxious substances used in colouring the sweetmeats known by the name of bonbons, and of which there is an immense consumption in France at the beginning of each year, has attracted the attention of the police; and, in consequence of discovering that the different colours were communicated by poisonous substances, great quantities have been destroyed. M. Chevalier states, that chromate of lead has been employed by some confectioners to give the yellow; the arsenite of copper, to give the beautiful green called Scheele's green; vermilion, or cinnabar in powder, for the red. (*Revue Medicale.*)

M. Piorry's *Pleximetre.*—M. Piorry has announced that, by means of a small pallet of ivory placed on any part of the abdomen or thorax, he has been enabled to turn percussion to a much better account than has yet been done; the sound emitted by the portions thus covered by the ivory pallet, being much clearer and more indicative of the actual state of the parts underneath, than when these parts are stricken by the fingers in the usual way.

M. Piorry has made more than eighty experiments on the living and dead subject, healthy and diseased. By means of this intervention, sound may be elicited from the thorax, even when the integuments are œdematous. The extent of the pleuritic effusions may be ascertained with great exactness. Hepatisation, and also a tuberculated condition of the lungs can be more readily detected in this way than by simple percussion. But it is in the abdominal region that the pleximetre promises to be of most service. The extent which any solid organ, as the liver, occupies—that of tumours—watery effusions, &c. may be easily ascertained by the difference of sound on percussion of the tablet, instead of the naked parietes. Even the quantity of fluid in the stomach or intestines can be appreciated. The state of the uterus (when pregnant) can be also correctly estimated through the medium of the pleximetre.

If discoveries go on in this manner for half a century more, there will be little occasion for the ancient desideratum—a glass window in the breast—to see what is going on within. (*Medico-Chirurgical Review.*)

*Peculiar Effects of Lightning.*—The returning stroke of lightning is well known to be due to the restoration of the natural electric state, after it has been disturbed by induction. Thus if a person be brought into a highly electric and negative state by induction, from the approximation of a body highly charged positively, and then the latter be discharged by means having no connexion with the negatively electrified person, the negative state of the latter will be immediately destroyed, and an effect, in part analogous to that of a positive discharge of electricity, will be produced. Some of the most serious accidents which occur from lightning are supposed to be produced in this way, not by the mere disturbance of electricity in a person only, but of the electricity of those bodies with which the person may be in contact, and to which he accidentally serves as a conductor.

On the 24th of Sept. 1826, at the moment when the lightning struck the ground, at the farm of Gali, near Versailles, M. B—— was violently affected by a returning stroke, at the distance of half a league from the place. The following are the circumstances of the case:—A violent storm occurred at Versailles and the neighbouring parts, at half-past nine o'clock. M. B., aged seventy-two years, was passing the Rue Dauphine, at a little distance from the church of Notre Dame, when one of those whirlwinds so common in the neighbourhood of large buildings, obliged him to turn round. He was then close to the party-wall of the houses 13 and 14, his right side being at a small distance from it. A metal water-pipe was fixed up the front of the house in this place, bringing the rain from the roof to the level of the pavement. In this position M. B. felt a commotion, which he describes as if all the right side of his body was roughly thrown towards the left, feeling, at the same time, much oppression, and vertigo, resembling that of drunkenness. The immediate effects were, difficulty of motion in all the left side, and a disturbed respiration; and it was with much difficulty, and only by resting frequently, that M. B. could reach the house of a neighbouring friend. It was there observed, that the tongue was embarrassed in its motions as well as the left side, but by the aid of attention the agitation of the mind was calmed; the night passed moderately, and the next morning all was nearly in its ordinary state. In the evening, however, at the hour when the circumstance occurred, all the symptoms returned, and the same results occurred daily until the end of the week, when a physician was consulted. He immediately recognized the symptoms of compression on the brain and spinal marrow, from which had resulted an incomplete paralysis of the tongue, and the left arm and leg. This speedily gave way under the hands of the physician, but the periodical returns occurred until the cure was completed.

It would be difficult to prove the identity of the electric discharge which fired the farm of Gali, and struck M. B., but the latter cannot be attributed to a direct stroke; for, at the moment when it happened, the intervals between the lightning and thunder were such as to show that the storm was not over Versailles. By a coincidence of circumstances, M. Demonferrand, who describes the case, was in the house No. 15, the whole of the evening, in an apartment contiguous to the metal pipe which appears to have served as a conductor for the electricity; but neither he nor any other person in the house felt the slightest disturbance. In the opposite house was a person in a bad state of health, and therefore, perhaps, more sensible to electric changes; but neither did he experience any change in his feelings at the moment. (*Jour. of Science.*)

*Sharp or Blunt Lancets in Vaccination.*—Dr. Gregory insists on the employment of a lancet ever sharper than one fit for venesection—but we know that a gentleman here who vaccinates an immense number annually, prefers a *broken* lancet. If the lancet be broken off about one-twelfth of an inch from the point, it may be pushed against the cuticle so as to abrade it without the loss of any blood, (which is the most frequent cause of failure, as the blood washes off the virus,) and then some of the dissolved virus is placed and pressed on the scratched part: this practice rarely fails. We have used the scab after keeping it several months; and, if carefully watched, the vesicle produced will, in such cases, be found strictly conformable to the character which the true vaccina ought to possess. (*North American Med. Journ.*)

*Prize Questions.*—The Society of Medicine at Caen propose the following prize questions:

Is Miliary Fever a disease *sui generis*, or only the result of a visceral irritation, or some other pathological state?

What are the principal diseases of which it may be a symptom, a complication, or crisis; and what are the modifications it may communicate or receive?

Have seasons, climates, localities, or even therapeutic measures, any influence on its development or severity?

To trace the history of miliary fever, and the best curative and prophylactic treatment.—The answers to these questions must be supported by clinical observations and anatomical researches.

The prize to be a gold medal, value 200 francs.

The essays must be sent free of postage, before the 31st December, 1827, addressed to M. Lafossa, fils, secretary of the Society. (*Rev. Med.*)

*Prize Questions.*—The Society of Practical Medicine of Paris propose the following questions:—

Question for 1827.—To determine, in the present state of science, the nature, seat, and treatment of scrofula?

Question for 1828.—To determine the different cases of disease where the employment of cold is useful, and those where it is dangerous; and to particularise the different modes of applying it?

The essays to be addressed, post-free, to M. Pascales, secretary general, Rue Chartereine, No. 36, before the 1st of November of each year.—The prize for each question, 300 francs. (*Ibid.*)

